# Growing demand for nursing in eye care in Sri Lanka

**Published:** 2020-12-31

**Authors:** Asela Abeydeera

**Affiliations:** 1 President: Association of Community Ophthalmologists of Sri Lanka.

**Sri Lanka is in need of a policy framework and a firm action plan to increase the number of trained ophthalmic nurses.**

**Figure:**
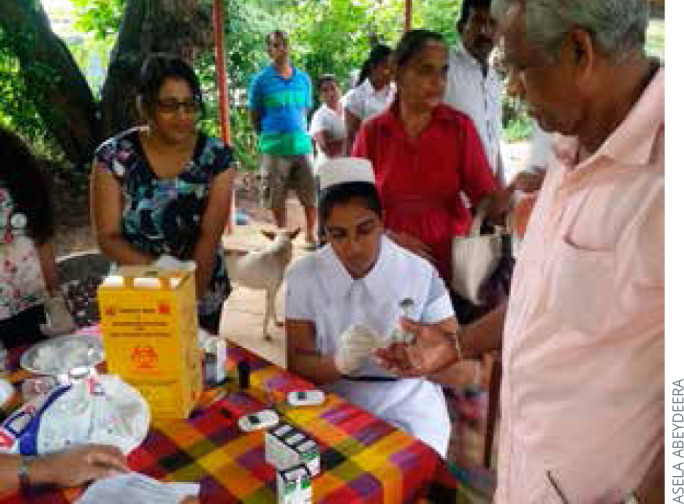
A nurse attends to a patient at Lions Eye Hospital, Ratnapura **SRI LANKA**

Sri Lanka is an island nation in the Indian Ocean, with a population of 21.5 million. It is identified as the fastest ageing nation in Southeast Asia. By the year 2050, one-third of the population in the country will be over 60 years of age.^1^ The prevalence of blindness among people over 40 years of age in Sri Lanka is 1.7%, and that of low vision is 17%. Over 150,000 people are estimated to be blind, and a further 1.5 million are estimated to have low vision.^2^ The ophthalmic services available are insufficient to address the eye care needs of Sri Lanka's population. The unavailability of local eye care services and the long distances people have to travel to seek eye care are significant barriers to access.

Ophthalmic nursing has become an important component of eye care globally, as it contributes towards the qualitative and quantitative improvement of the care provided. The roles and responsibilities of ophthalmic nurses vary from country to country.^3^ In some countries, they are engaged in health promotion, prevention and intervention, while other countries have restricted their services.

The history of nursing in eye care in Sri Lanka starts in the early years of the last century, with the establishment of Colombo Eye Hospital in 1905. Most ophthalmic nurses in Sri Lanka work at eye hospitals and clinics.

There are three main eye health sectors in Sri Lanka where ophthalmic nursing is practiced:

1. Government and semi-government eye hospitals and eye units (Free-of-cost service)
2. Charity, partial charity, and not-for-profit eye hospitals
3. Private sector eye hospitals and departments.

**Figure 2:**
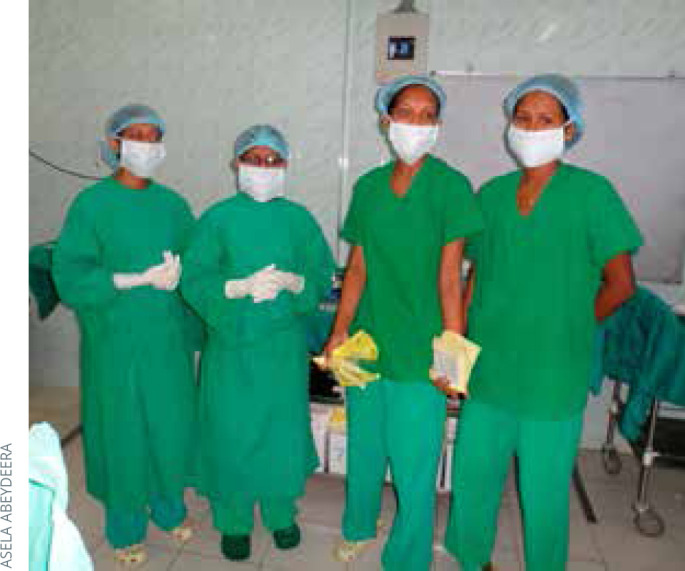
Nurses at Lions Eye Hospital in Colombo. **SRI LANKA**

However, there are no specific ophthalmic nurse training programmes within the ministry of health. Nurses in the first sector are trained at government general nursing schools operated by the ministry of health and at the government universities in Sri Lanka.^4^ The diploma course takes two years, and the degree course takes three years. However, the nurses gain limited knowledge in eye care during their studies, as just two weeks are allocated to eye care.

Following graduation, nurses are posted to different hospitals. Those who are posted to eye units and eye hospitals receive hands-on training in addition to a few workshops conducted at their centres or at a central level. The type of training they receive, e.g., eye clinic practice, operating theatre procedures, or ward work, largely depends on their place of work. Structured continuing medical education programmes and postgraduate training in ophthalmic nursing are still not available in the country.

Nurses for the not-for-profit and private hospitals are trained in the nursing schools operating under the private sector. These institutions recruit nurses with a certificate in nursing, such as a diploma, and not a degree. They train them in-house, according to the requirements of the hospitals.

Ophthalmic nurses take on various roles, such as:

- nursing manager or nursing sister
- eye clinic nurse
- operating theatre nurse
- ward nurse
- some nurses are given particular tasks, such as health education, infection control, community work, etc.

Colombo National Eye Hospital is the largest eye care centre in Sri Lanka, with over 400 beds. As a centre of excellence, the hospital caters to the whole country through different ophthalmic sub-specialities. Over 150 nurses are employed at this hospital, which has a very high volume of patients. There are over 50 smaller eye units across the country, they are affiliated to government hospitals, and have fewer nurses assigned to them.

Ophthalmologists have noticed that the nurses have limited knowledge and training in anatomy, physiology and ophthalmic pharmacology. Therefore, the College of Ophthalmologists of Sri Lanka conducts periodic training and orientation programmes for them. The institutes for the nurses in Sri Lanka also conduct short refresher education programmes in clinical nursing. Despite these efforts, there is still a need for a structured postgraduate education in ophthalmic nursing in Sri Lanka.

There are five non-governmental charity hospitals for eye care in Sri Lanka (operated by Lions Club and HelpAge Sri Lanka), and dozens of private hospitals. All these institutions provide secondary and tertiary level eye care. Eye hospitals operated by Lions Clubs are involved in running occasional community eye care programmes, usually in remote areas. They use nurses as screeners and counsellors.

Overall, the available human resources in eye care are not sufficient to cater to the needs of the nation. In this context, the role of an ophthalmic nurse as a primary eye care worker is vital. As part of the VISION 2020 programme, during its early years, training and orientation programmes were conducted for public health midwives (PHM) in Sri Lanka.^5^ Since PHMs are grass-root level health care workers, and are mainly involved in maternal and child health, they find it difficult to cope with the extra responsibilities assigned to them, including eye care.

The need for dedicated primary eye care workers/nurses is well recognised. Successful primary eye care models need to be initiated to meet the growing need for eye care services in rural areas. Similar successful models are practised in other countries. For example, Aravind Eye Care System in India has proved the efficiency of utilising ophthalmic nurses at their vision centres. Rwanda showed encouraging results by engaging trained ophthalmic nurses in primary eye care.^6^ During recent years, the ministry of health of Sri Lanka has started the exercise of assigning nurses to public health institutions, but without incorporating any specific eye care component.

The following measures are highly recommended for Sri Lanka, with regard to clinical and community-based ophthalmic nursing services, in order to achieve better eye care services, and make them accessible to all:

- Advocate for policy regarding the importance of nursing in eye care and nurses' potential to improve eye care service delivery.
- Increase the number of nurses recruited in the government sector.
- Include primary eye care as an essential component in nursing curricula.
- Develop and deliver hands-on training in relevant areas in ophthalmology that the nurses can use in their jobs. This can be in-service training or continuing medical education-type learning processes.
- Recruit adequate number of trained ophthalmic nurses at primary health care institutions.
- Establish an effective referral system, initiated from the community eye nurse to the secondary and tertiary level eye care service providers.
- Follow up patients who need long-term care with the help of community eye nurses. Use a dedicated mechanism to do so.

**Figure 3:**
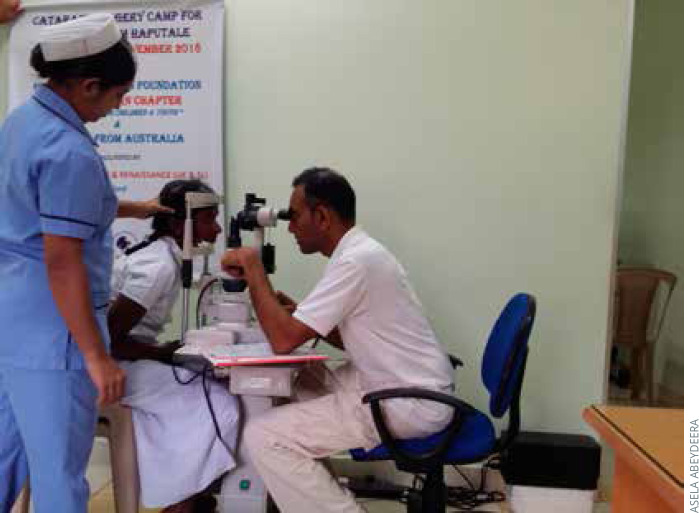
An eye camp in the district of Badulla. **SRI LANKA**
